# Analgesic and anti-inflammatory activity of amifostine, DRDE-07, and their analogs, in mice

**DOI:** 10.4103/0253-7613.62401

**Published:** 2010-02

**Authors:** Yangchen Doma Bhutia, Rajagopalan Vijayaraghavan, Uma Pathak

**Affiliations:** Defence Research and Development Establishment, Gwalior, India

**Keywords:** Amifostine sulfur, analgesic activity, anti-inflammatory activity, DRDE-07, sulfur mustard

## Abstract

**Objectives:**

To find out the analgesic and anti-inflammatory activity, if any, of Amifostine [S-2(3 amino propyl amino) ethyl phosphorothioate], DRDE-07 [S-2(3 amino ethyl amino) ethyl phenyl sulphide] and their analogs DRDE-30 and DRDE-35, the probable prophylactic agent for sulphur mustard (SM).

**Materials and Methods:**

In order to find out the analgesic activities of the compounds two methods were employed, namely, acetic acid-induced writhing test and formalin-induced paw licking. The persistent pain model of formalin-induced hind paw licking was carried out to test the effect of the compounds on neurogenic pain or early phase (0 to 5 minutes) and on the peripheral pain or the late phase (15 to 30 minutes). To test the effect of the compound in acute inflammation, carrageenan-induced hind paw edema was carried out. This model of inflammation involves a variety of mediators of inflammation.

**Results:**

DRDE-07 (81.7%) and DRDE-30 (79.4%) showed significant reduction in the acetic acid-induced writhing test. DRDE-07 (93.1%), DRDE-30 (82%), and DRDE-35 (61.3%) showed significant reduction in the second or late phase of formalin-induced paw licking. All the analogs (more than 60%) including amifostine (43.9%) showed significant reduction of paw edema in the carrageenan-induced paw edema in mice.

**Conclusion:**

The analgesic and anti-inflammatory activity of the antidotes were comparable with aspirin.

## Introduction

Bis (2-chloroethyl) sulphide, a chemical warfare agent, commonly known as sulphur mustard (SM) is a powerful blistering and vesicating agent. It is thought to form a sulphonium ion in the body, which alkylates DNA.[1] After years of research, there is still no satisfactory antidote that has been recommended to completely protect against sulphur mustard toxicity. Numerous approaches have been tried such as, personnel decontamination, prevention of alkylation, and retrieval of SM-alkylated DNA, but they have not been very successful.[2] The hallmarks of SM toxicity are edema, dermal infiltration of inflammatory cells, and the premature death of basal epidermal cells. Therefore, early therapeutic intervention after SM exposure, with anti-inflammatory and analgesic compounds could alleviate all the downstream events.

Many studies have been carried out by researchers that point to the importance of the use of anti-inflammatory drugs against SM. Topical application of adexone, a steroid, and voltaren, a non-steroidal anti-inflammatory agent was found to be effective in decreasing SM-induced injury.[3] Systemic administration of anti-inflammatory drugs, such as, indomethacin, olvanil, and hydrocortisone, significantly reduced the edema induced by SM.[4,5] Pre-treatment with indomethacin, in one study, was found to reduce SM-induced damage.[6] Anti-inflammatory drugs reduce the dermal, respiratory, and ocular damage caused by exposure to SM.[7] Amifostine, a radio-protective agent and its analogs have shown protection against SM toxicity *in vivo* and *in vitro*.[8,11] These compounds may have numerous mechanisms by which they may be imparting protection against SM. Inflammatory changes in SM-induced toxicity have been reported and it starts even before all other changes can be detected. As amifostine, DRDE-07, DRDE-30, and DRDE-35 give remarkable protection against SM, the present study is undertaken to explore their analgesic and anti-inflammatory activity, if any.

## Materials and Methods

**Chemicals:** DRDE-07, DRDE-30, DRDE-35, and Amifostine were synthesized in the Synthetic Chemistry Division of the Establishment. The compounds were characterized by elemental analysis, IR, ^1^HNMR, and mass spectral analysis. The purity was checked by thin layer chromatography (TLC). The test compounds were of 99% purity.[10] Aspirin and carrageenan were obtained from Sigma Aldrich. All other chemicals used were of analytical grade.

**Animals:** Swiss albino mice were obtained from the animal facility of the Establishment. The animals were housed in polypropylene cages with sterilized rice husk as bedding material. They were fed with pelleted diet from Ashirwad Feed, (India), and water was provided *ad libitum*. The care and maintenance of the animals were as per the approved guidelines of the Committee for the Purpose of Control and Supervision of Experiments on Animals in India. The study was the approved by the Institutional Animal Ethical Committee.

**Grouping of animals:** Three experiments were carried out:

- Acetic acid-induced writhing
- Formalin-induced paw licking
- Carrageenan-induced hind paw edema

For each experiment six groups consisting of six animals were used.

Group I served as the control and received comparable amount of vehicle (distilled water). aspirin, 300 mg/kg p.o., amifostine, 186 mg/kg p.o., DRDE-07, 249 mg/kg p.o., DRDE-30, 219 mg/kg p.o., and DRDE-35, 230 mg/kg p.o. were administered to animals of groups II, III, IV, V, and VI, respectively.

### Analgesic activity

**Acetic acid-induced writhing in mice:** The experiment was carried out according to the method of Witkin *et al*[12] Drugs were administered by the oral route one hour prior to the injection of acetic acid. Writhing was induced in animals by injecting acetic acid 300 mg/kg (3% solution in sterile distilled water) i.p. Each mouse was then put into a big glass cylinder and the total number of writhing episodes for a period of 20 minutes after the injection of acetic acid was counted. The percent inhibition of the writhing count of the treated group was calculated from the mean writhing count of the control group.

**Formalin-induced hind paw-licking:** The formalin-induced hind-paw licking was performed as per the method described by Hunskar and Hole.[13] One hour after drug administration by the oral route, formalin was injected (2.5%, 20 μl) sub-plantar, in the right hind paw, and the duration of paw licking as an index of nociception was counted in periods of 0 to 5 minutes (early phase) and 15 to 30 minutes (late phase). The left paw was injected with sterile phosphate buffered saline.

### Anti-inflammatory activity

**Carrageenan-induced hind paw edema in mice:** This test was conducted as per the method described by Winter *et al*.[14] The mice were fasted for 16 hours, but water was allowed *ad libitum*. The paw thickness (0 hour) was measured, in millimetres, using digital vernier callipers (Mitutoyo, Japan). The test substances and standard drug were administered one hour prior to the injection of a phlogistic agent via the oral route. The phlogistic agent carrageenan was prepared as 1% suspension, in sterile normal saline, a day before the study, to get a proper suspension. Carrageenan 0.1 ml was injected subcutaneously into the right hind paw of each mouse. The thickness of the injected paw was measured three hours later. The edema thickness (mm) was calculated by subtracting the zero-hour reading from the three-hour reading. From the mean edema volume, the percentage inhibition of the edema was calculated between the treated and control groups.

Percent (%) inhibition = V_C_ − V_t_ × 100 / V_C_

Where, V_C_ and V_t_ represent the average paw volume in the control and treated groups, respectively.

### Statistics

The results were analyzed by One Way Analysis of Variance followed by the Student Newman Keul's test. The probability of 0.05 or less was considered statistically significant. For the statistical analysis Sigma Stat (SPSS Inc, USA) was used.

## Results

### Acetic acid-induced writhing

**Figure 1:**
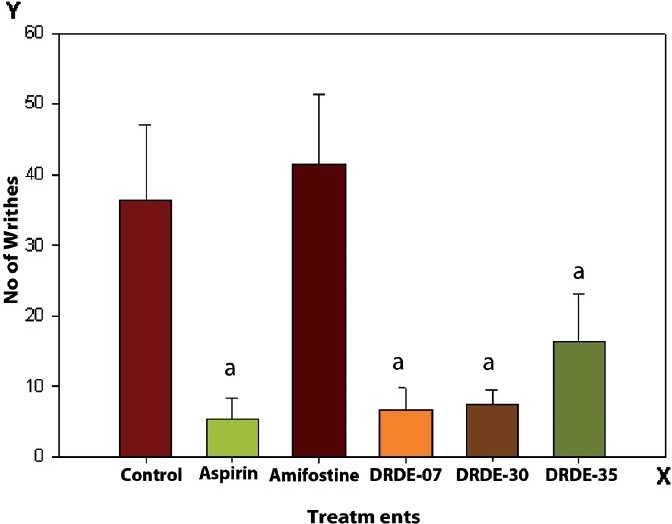
Acetic acid-induced writhing in mice (Mean ± SEM, 6 replicates) [The drugs / chemicals were administered one hour prior to the acetic-acid injection, as a single oral dose of aspirin (300 mg/kg); DRDE-07 (249 mg/kg); DRDE-30(219 mg/kg); DRDE 35(230 mg/kg)] a = significantly different from the control

The results are shown in Figure 1. The writhing counts decreased significantly in the group treated with DRDE-07 to about 81.7% inhibition, which was comparable to the standard drug aspirin having 85.3% inhibition. The groups treated with DRDE-30 and DRDE-35 showed significant reduction in writhing due to acetic acid, the percent inhibition being 79.4 and 54.7 respectively. The group treated with amifostine did not show any significant change.

### Formalin-induced paw licking

**Figure 2:**
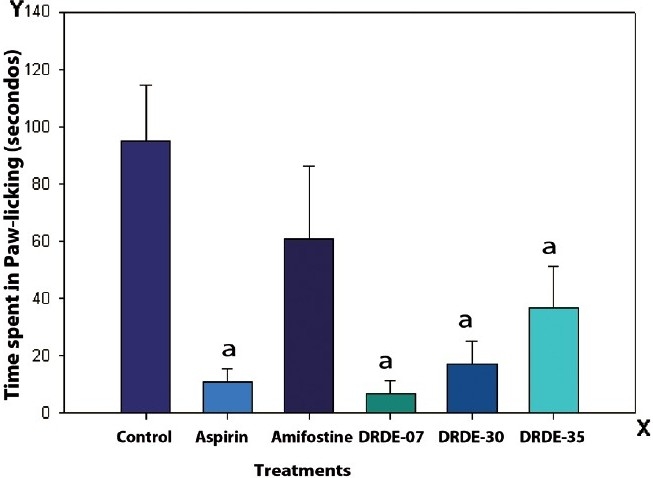
Formalin-induced paw licking (in seconds), second phase in mice (Mean ± SEM, 6 replicates) [The drugs / chemicals were administered one hour prior to the formalin injection, as a single oral dose of Aspirin (300 mg/kg); DRDE-07 (249 mg/kg); DRDE-30(219 mg/kg); DRDE 35(230 mg/kg)]. a = significantly different from the control

The paw licking phases were divided into two parts, the early phase (0 to 5 minutes) and the late phase (15 to 30 minutes). In the early phase there was reduction in the number of episodes of licking, but there was no significant difference between the control and any of the treatment groups. In the second phase DRDE-07 showed a significant decrease in paw licking. The percent inhibition of licking was 93.1, which was lower than the group treated with standard drug aspirin, having a percent inhibition of 88.7. The groups treated with DRDE-30 and DRDE-35 also showed a significant reduction in paw licking, the percent inhibition being 82 and 61.3, respectively [Figure 2]. Amifostine has a partial effect on the inhibition of paw licking, with the percent inhibition of paw licking being 36.3.

### Carrageenan-induced paw edema

**Figure 3:**
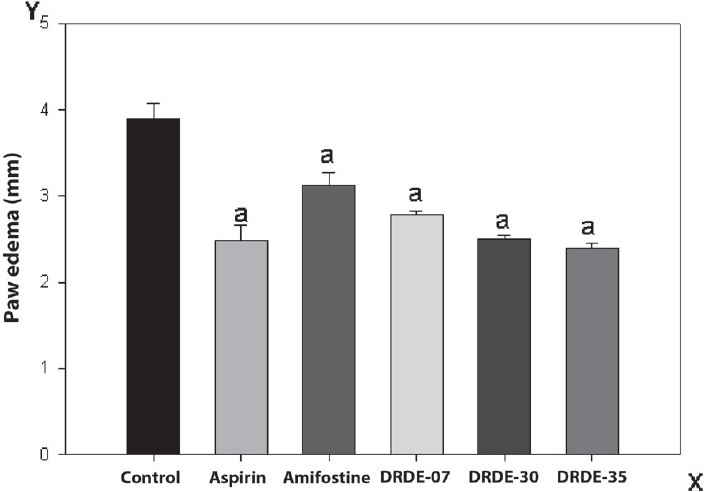
Carrageenan-induced paw edema in mice (in mm); (Mean ± SEM, 6 replicates) [The drugs / chemicals were administered one hour prior to the carrageenan injection, as a single oral dose of Aspirin (300 mg/kg); DRDE-07 (249 mg/kg); DRDE-30(219 mg/kg); DRDE 35(230 mg/kg)]. a= significantly different from the control

The results are shown in Figure 3. The paw edema showed significant reduction in the group treated with standard drug aspirin and also with the other treatment groups. The percentage inhibition was 83.62, 43.85, 60.23, 67.83, and 78.36 for aspirin, amifostine, DRDE-07, DRDE-30, and DRDE-35, respectively.

## Discussion

Amifostine, DRDE-07, and their analogs have shown remarkable protection against sulphur mustard injury systemic toxicity.[8–10] In this study we have evaluated the analgesic and anti-inflammatory effect of these compounds. The results show that the analgesic and anti-inflammatory effects of these compounds are comparable to those of aspirin.

The peripheral analgesic effect was tested by acetic acid-induced writhing test in mice. The reference drug used was aspirin, as it offers relief from inflammatory pain, by inhibiting the formation of pain mediators in the peripheral tissues, where prostaglandins and bradykinins are said to play a significant role in the pain process. Acetic acid-induced writhing is a standard test for pain sensitivity to opiates as well as to non-opiate analgesics.[15,16] The associated nociceptive response is believed to involve the release of endogenous substances such as bradykinin and prostanoids, among others, which stimulate the nociceptive endings.[17] DRDE-07 and the analogs have shown good analgesic activity in this model and have produced a significant decrease in the writhing counts.

The persistent-pain model of formalin-induced hind paw licking was used in the study. The first phase of pain is attributed to the direct activation of nociceptors and primary afferent fibers by formalin, causing the release of bradykinin and tachykinins.[18,19] This phase is inhibited by opioid analgesics.[20] The second phase is due to an inflammatory reaction caused by tissue injury leading to the release of histamine, serotonin, prostaglandin, and excitatory amino acids.[21,22] This late phase is inhibited by non-steroidal, anti-Inflammatory drugs (NSAIDs) and opioid analgesics. DRDE-07, DRDE-30, and DRDE-35 significantly decrease the paw licking time in the second phase. This indicates an NSAID-like action. Aspirin, as expected, exhibits a significant analgesic activity only in the second phase. Our finding is substantiated by reports that aspirin exhibited greater anti-nociceptive activity in this model of inflammatory pain.

Both steroidal and non-steroidal anti-inflammatory drugs can be tested by the carrageenan-induced paw inflammation test. The edema induced in the mouse paw by the injection of 1% carrageenan is brought about by autacoids, histamine, and 5-hydroxy tryptamine (5-HT) during the first one hour, after which the kinins act, to increase the vascular permeability up to two-and-a-half hours. The maximum inflammation is seen approximately three hours post the carrageenan injection, after which it begins to decline.[23] Following that, the prostaglandins act from two and a half hours to six hours, which results in the migration of leucocytes into the inflamed site.[24,25] DRDE-07 and the analogs show a significant inhibition of inflammation, which is comparable to standard drug aspirin.

All the compounds such as amifostine, DRDE-07, DRDE-30, and DRDE-35 have shown potent analgesic and anti-inflammatory activity, which may also contribute to the protection against SM toxicity.
